# Supplementary material for: Group 2i Isochrysidales produce characteristic alkenones reflecting sea ice distribution
Source: Nat Commun. 2021 Jan 4;12:15. doi: 10.1038/s41467-020-20187-z (PMC7782803; doi:10.1038/s41467-020-20187-z)
Supplement: Supplementary file 1 — Supplementary Information [file 41467_2020_20187_MOESM1_ESM.pdf]

Supplementary Information for *Group 2i Isochrysidales produce characteristic alkenones reflecting sea ice distribution* by Wang et al.

Karen Jiaxi Wang[1,2,*], Yongsong Huang[1,2,*], Markus Majaneva[3], Simon T. Belt[4], Sian Liao[2,5], Joseph Novak[1], Tyler R. Kartzinel[2,6], Timothy D. Herbert[1,2], Nora Richter[1,2,7], Patricia Cabedo-Sanz[4]

[1] *Department of Earth, Environmental and Planetary Sciences, Brown University, Providence, RI 02912, USA*

[2] *Institute at Brown for Environment and Society, Brown University, Providence, RI 02912, USA*

[3] *Norwegian Institute for Nature Research (NINA), Trondheim, NO-7485, Norway*

[4] *Biogeochemistry Research Centre, School of Geography, Earth and Environmental Sciences, Plymouth University, Plymouth, PL4 8AA, UK*

[5] *Department of Chemistry, Brown University, Providence, RI 02912, USA*

[6] *Department of Ecology and Evolutionary Biology, Brown University, Providence, RI 02912, USA*

[7] *Department of Marine Microbiology and Biogeochemistry, NIOZ Royal Netherlands Institute for Sea Research, Texel, The Netherlands*

* Corresponding Authors:

E-mail: yongsong_huang@brown.edu (Yongsong Huang) and karen_wang@brown.edu (Karen Wang)

Tel: +1 401 863 3822

Fax: +1 401 863 205

Declarations of interest: none

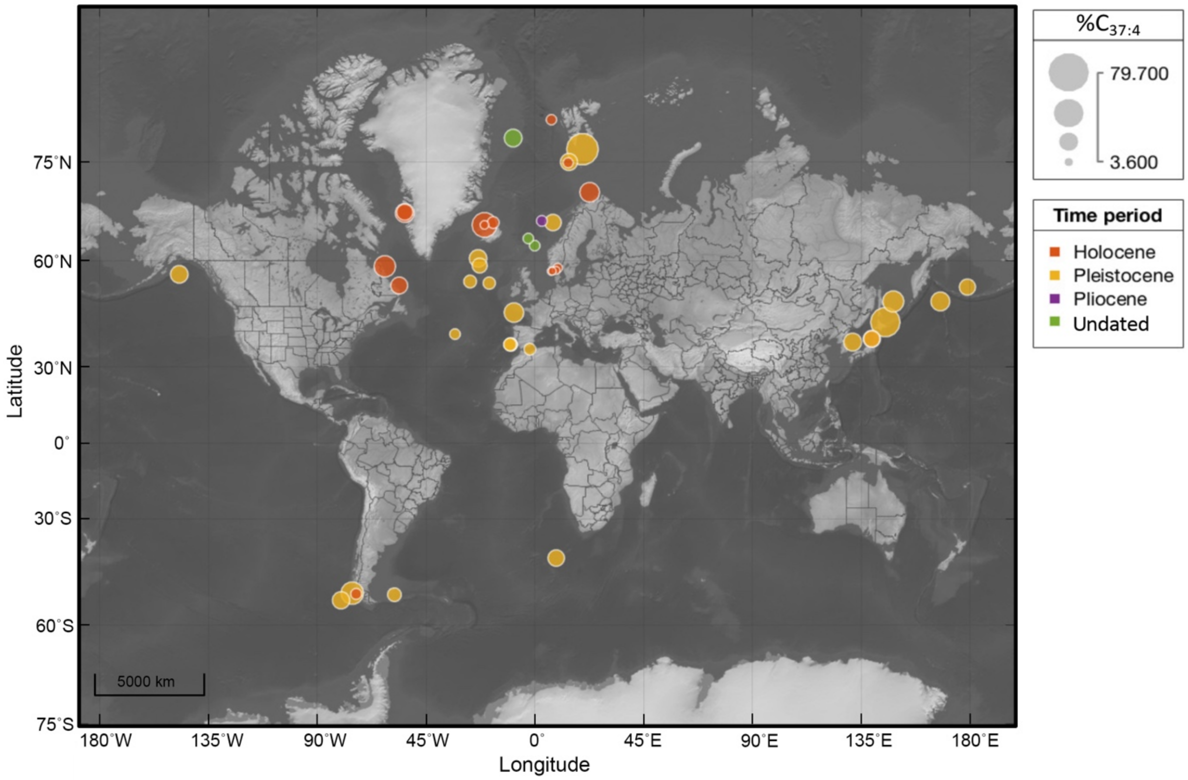

Supplementary Figure 1. Compilation of reported %$C_{37:4}$ records listed in **Supplementary Table 1**. The bubble size represent highest %$C_{37:4}$ value reported in the record, and the color represent the time period of the highest value reported.

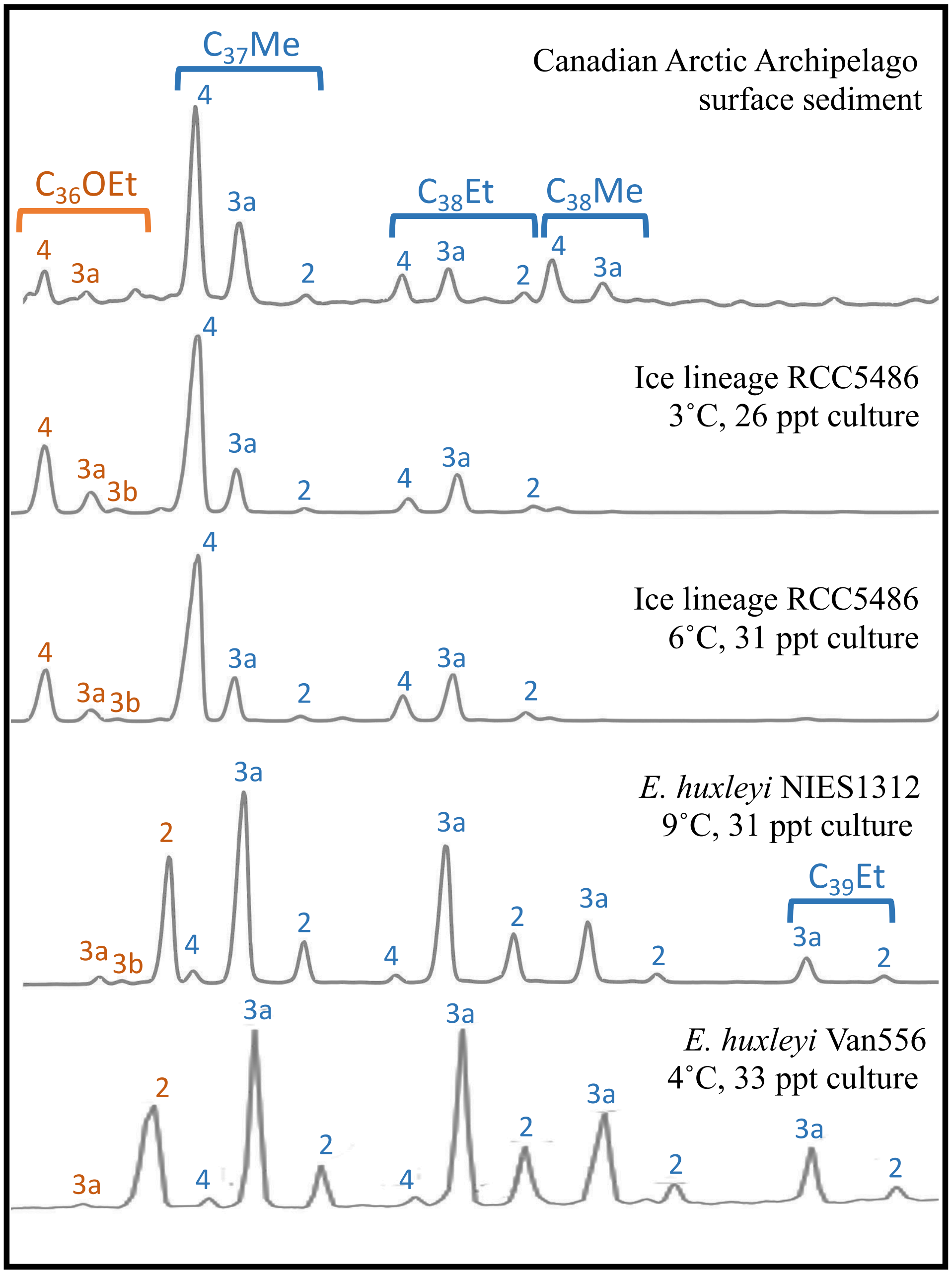

Supplementary Figure 2. Gas chromatograms showing alkenone and alkenoate profiles generated from Canadian Arctic Archipelago surface sediments, RCC5486 cultures, *E. huxleyi* strain NIES1312 (Liao et al., unpublished) and Van 556 cultures[1]. C_n represent the number of carbon; Me = methyl ketone, Et = ethyl ketone, OEt = ethyl alkenoate; number above each peaks indicate the number of double bonds; 3a and 3b are tri-unsaturated double bond positional isomers.

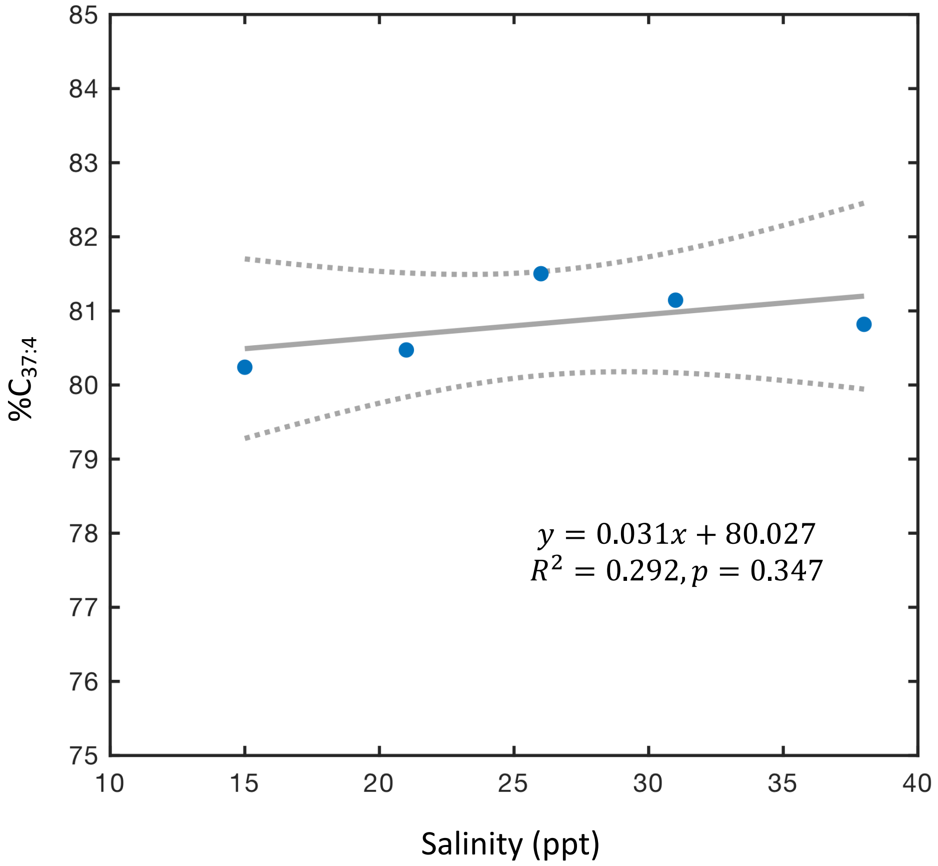

Supplementary Figure 3. $\%C_{37:4}$ produced by RCC5486 under 3˚C does not show any significant correlation with salinity. Each data point represent the averaged value of the triplicated culture experiments. The dashed lines denote 95% confidence interval for the regression.

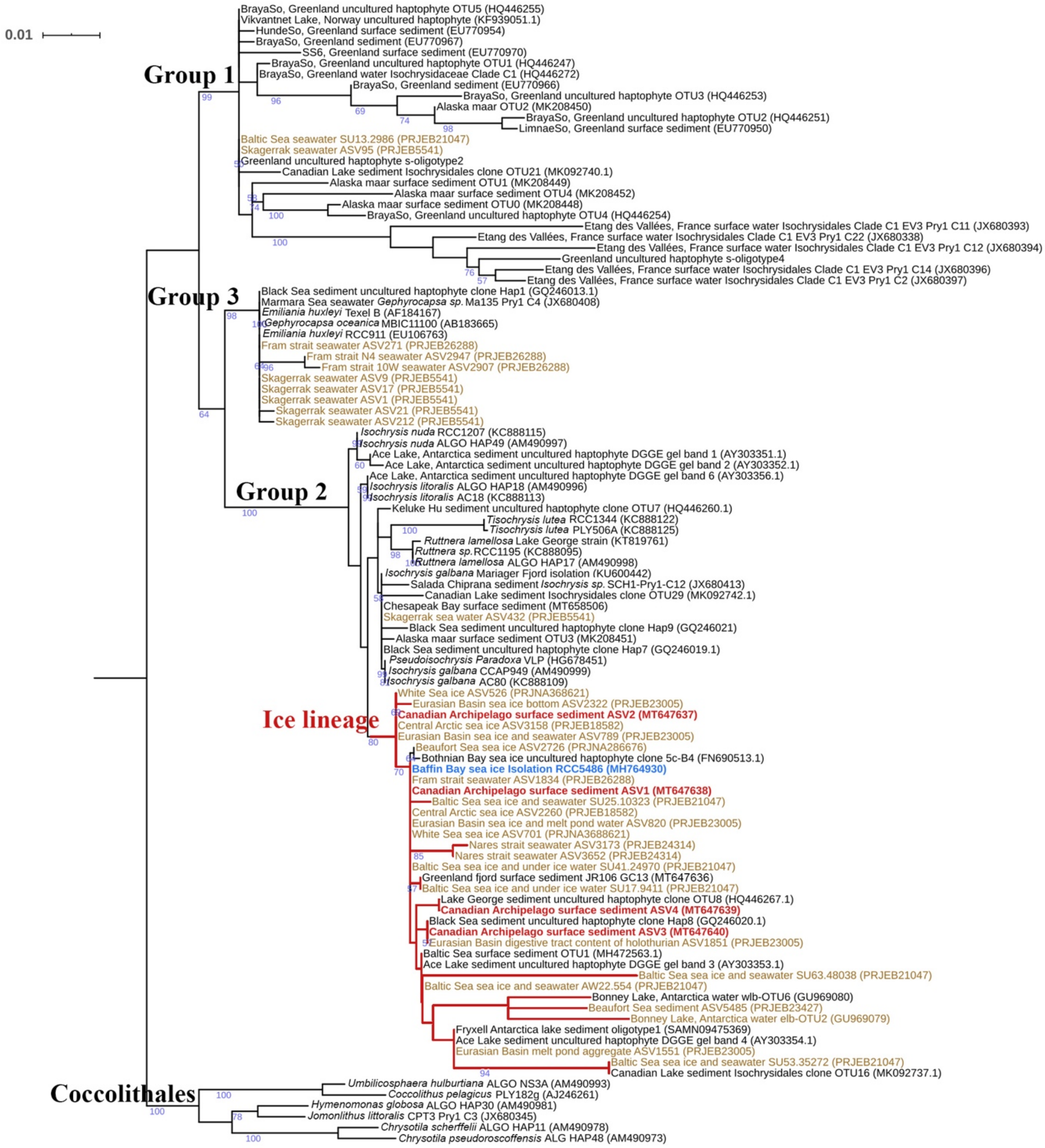

Supplementary Figure 4. The full phylogenetic tree showing the position of the novel ice-lineage Isochrysidales. This maximum-likelihood tree is based on 18S rRNA gene from 99 sequences spanning three groups of Isochrysidales and 6 from the Coccolithales outgroup. The numbers shown on branches represent % bootstrap support for the node (only those >50% are shown). The scale bar represents substitutions per site. Sequences generated from Canadian Arctic Archipelago surface sediments in this study are highlighted in red, and sea-ice isolated strain RCC5486 cultured in this study is highlighted in blue font. Sequences from re-analyzed NGS datasets are indicated by brown font, and other sequences were obtained from NCBI GenBank. The branches of the ice-lineage Isochrysidales are highlighted in red. A version of this tree with collapsed nodes for easy viewing is provided in **Figure 1** of the main text.

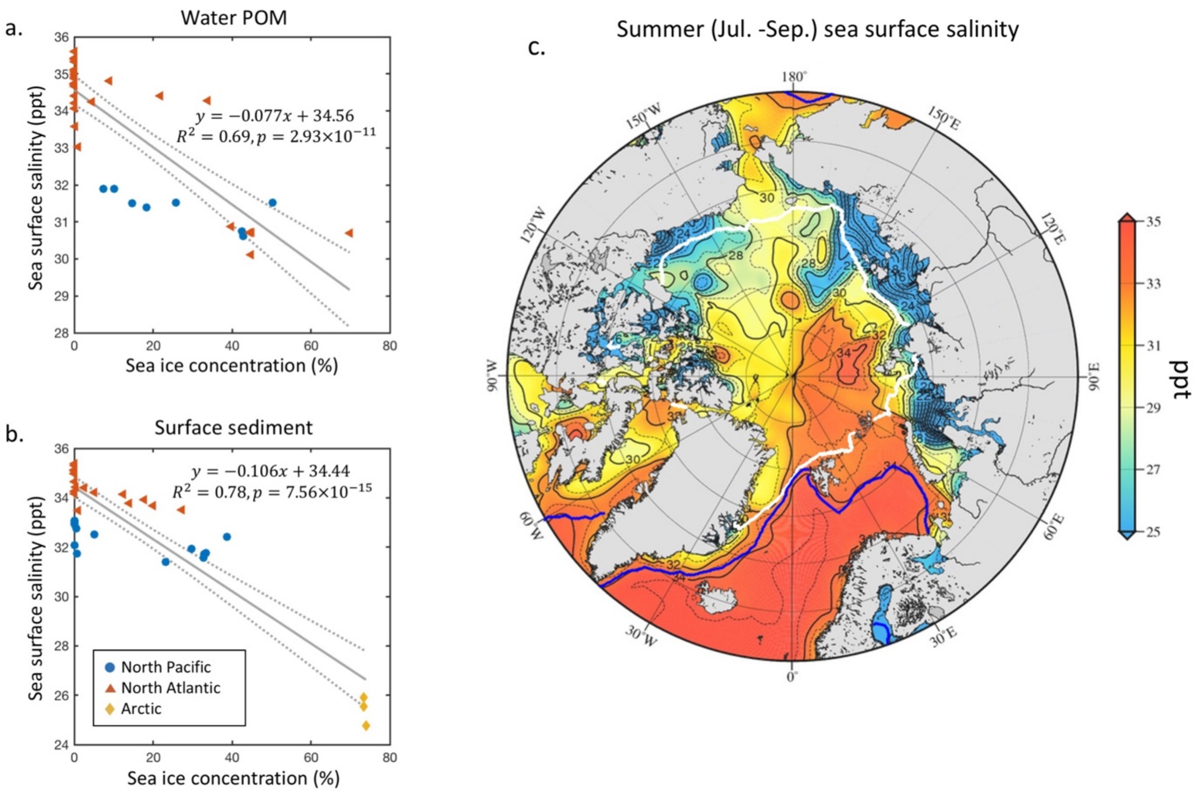

Supplementary Figure 5. a) Linear regression between mean sea ice concentration 12-month prior to POM sample collection and measured SSS during sample collection compiled from previous studies[2,3] (n=40 independent samples). b) Linear regression between 1981-2010 annual mean sea ice concentration and 1981-2010 annual mean SSS[4] at surface sediment sites in this study (n=42 independent samples). The dashed lines denote 95% confidence interval for the regression in a) and b). c) shows modern summer (Jul.- Sep.) SSS in the circum-Arctic region adapted from https://www.nodc.noaa.gov/OC5/regional_climate/arctic/, with the blue and white lines indicating March and September median sea ice extent respectively (NSIDC).

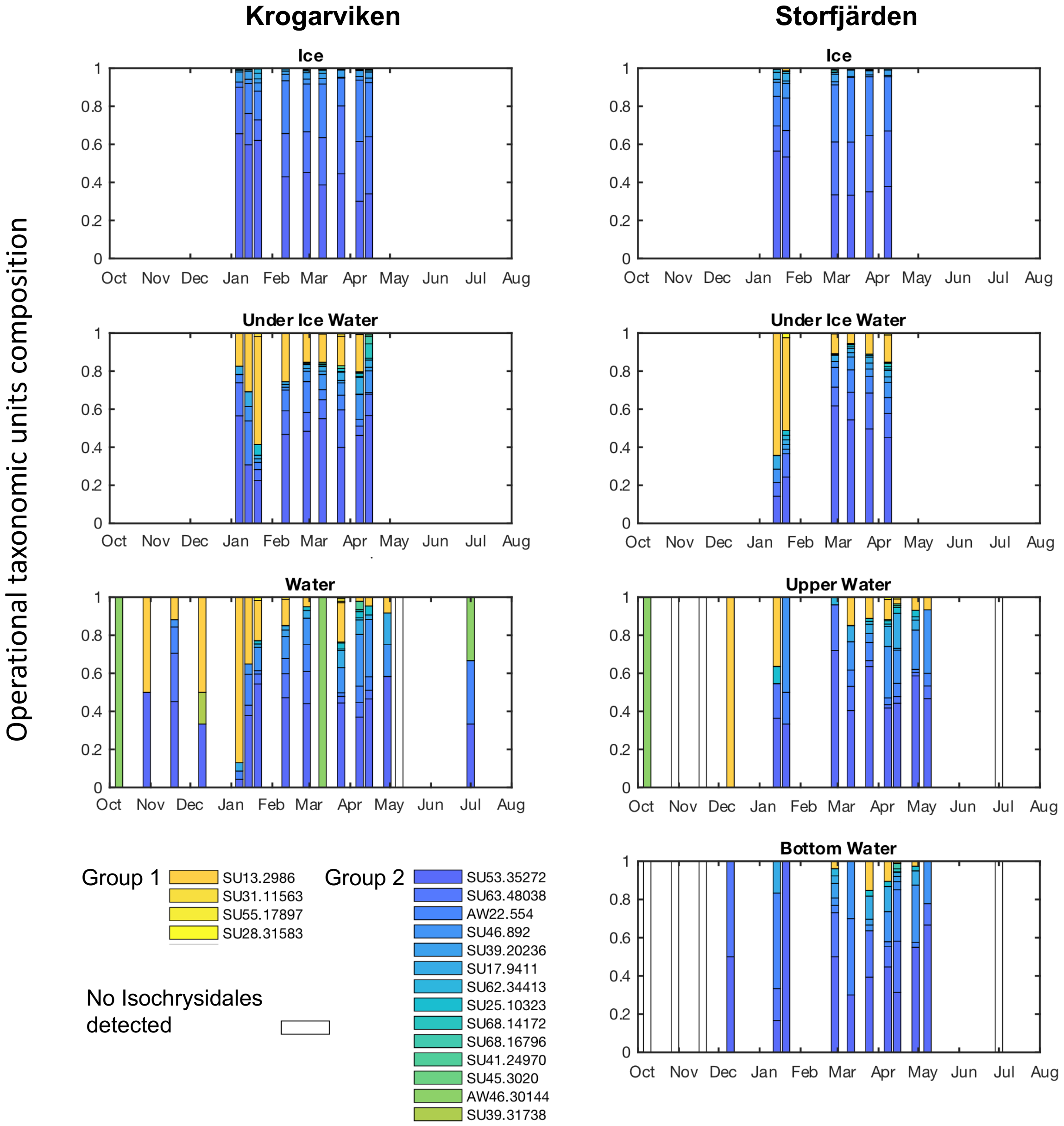

Supplementary Figure 6. Seasonal succession of Isochrysidales from Autumn to Spring in the northwest of Gulf of Finland, Baltic Sea, from re-analyzed NGS data[5]. OTU composition (% of OTU reads to total Isochrysidales reads) in ice, under ice water, 0-3m water, 3-15m water at Krogarviken (59° 50.650’ N, 23° 15.100’ E) and Storfjärden (59° 51.250’ N, 23° 15.815’ E). Notably, Group 2i are the only Isochrysidales species detected within the ice in Krogarviken and compose >99% reads within the ice in Storfjärden; Group 1 species are mostly found in water samples with only a few reads (1-3) detected within ice samples from Storfjärden.

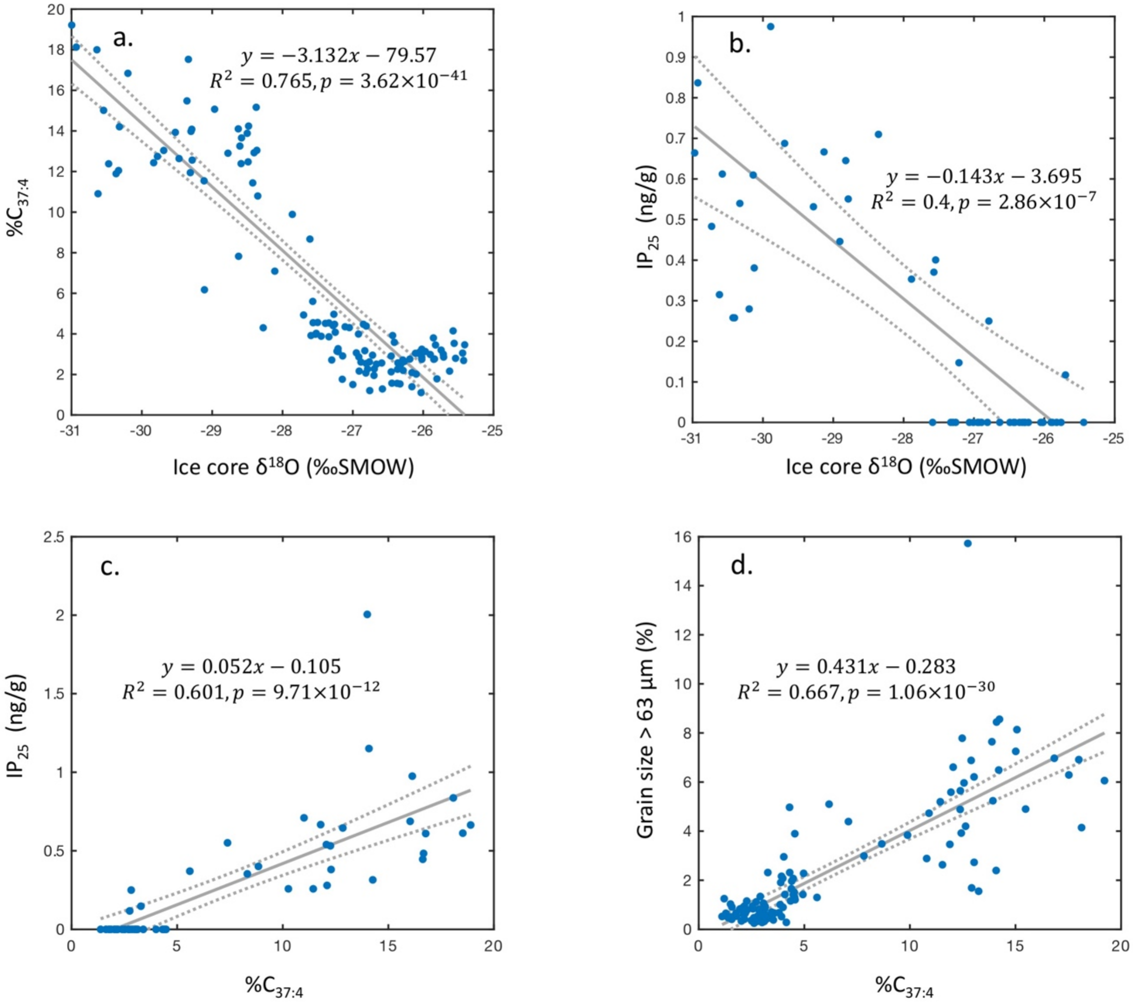

Supplementary Figure 7. a) Linear regression between $\%C_{37:4}$ from M23258-2[6] and $\delta^{18}O$ from Renland ice core[7] (n=127 independent samples). b) Linear regression between IP_{25} from M23258-2 and $\delta^{18}O$ from Renland ice core[7] (n=54 independent samples). c) Linear regression between $\%C_{37:4}$[6] and IP_{25} in core M23258-2 (n=53 independent samples). d) Linear regression between $\%C_{37:4}$[6] and >63 µm grain size[8] in core M23258-2 (n=123 independent samples). The dashed lines denote 95% confidence interval for the regression.

Supplementary Table 1. List of published %$C_{37:4}$ sediment-core records shown in **Supplementary Figure 1** and the paleoclimate interpretations.

Core	Reference	Region	Published interpretation	Lat.	Lon.	Highest %$C_{37:4}$	Record length
GS15-198-33	Perner et al.[9]	north Icelandic shelf	freshwater flux	66.6	-20.9	5	1850-2014 AD
MSM05/05-712-1	Rueda et al.[10]	Fram Strait	colder and fresher water	78.9	6.8	8	0-2000 AD
MSM45-31-1	Lochte et al.[11]	Labrador Shelf	sea-ice or meltwater	54.4	-56.0	22	0-6.2 kyr BP
372610	Krossa et al.[12]	west Skagerrak	low salinity Baltic outflow	57.7	6.7	4	0-4 kyr BP
225514	Krossa et al.[12]	central Skagerrak	low salinity Baltic outflow	57.8	8.7	4	0-4 kyr BP
372650	Krossa et al.[12]	northeast Skagerrak	low salinity Baltic outflow	58.5	9.6	6	0-5 kyr BP
242940	Krossa et al.[12]	west Skagerrak	low salinity Baltic outflow	57.7	7.2	4	0-4 kyr BP
343310	Moros et al.[13]	west Greenland	low SSS due to meltwater input	68.6	-53.8	20	0-3.5 kyr BP
343300	Moros et al.[13]	west Greenland	low SSS due to meltwater input	68.5	-54.0	28	0-8 kyr BP
SV04	Rigual-Hernández et al.[14]	north eastern Norwegian Sea	-	75.0	13.9	7	0.73-10.49 kyr BP
MSM45-19-2	Lochte et al.[15]	Labrador Shelf	sea-ice or meltwater	58.8	-61.9	36	0-8.9 kyr BP
JR51-GC35	Bendle et al.[16]	north Icelandic shelf	polar water mass	67.0	-17.2	10	0.06-10.17 kyr BP
PC03	Harada et al.[17]	Chilean Patagonia fjords	low salinity	-52.9	-74.1	9	0-12 kyr BP
PC-9	Ishiwatari et al.[18]	Japan Sea	potentially low salinity	39.6	139.4	23	1.0-36 kyr BP
MD99-2269	Kristjánsdóttir et al.[19]	Icelandic shelf	potentially low salinity	66.6	-20.9	49	0.4-11.38 kyr BP
Psh-5159	Risebrobakken et al.[20]	south western Barents Sea	freshwater influence	71.4	22.7	30	0-11.42 kyr BP
JM-09-020	Łącka et al.[21]	Storfjordrenna, west Barents Sea	polar water mass	76.3	19.7	80	1.29-13.99 kyr BP
M23258-2	Martrat et al.[6]	north eastern Norwegian Sea	polar water mass	75.0	14.0	23	0.76-14.12 kyr BP
MD95-2011	Calvo et al.[22]	NE Norwegian Sea	polar water mass (colder and fresher)	67.0	7.6	21	0.52-15 kyr BP
MD09 2002	Ménot et al.[23]	Bay of Biscay	low salinity from iceberg from British-Irish ice sheet	47.5	-8.5	30	5.0-30 kyr BP
MD07-3128	Caniupán et al.[24]	Chilean Patagonia fjords	low salinity	-52.7	-75.6	40	0-60 kyr BP
JR244-GC528	Roberts et al.[25]	Drake passage	indicating alkenone-SST bias	-53.0	-58.0	14	1-25.44 kyr BP

M23415	Rosell-Melé et al.[26]	North Atlantic	freshening of surface waters in response to incoming freshwater from sea-ice and/or icebergs	55.0	-19.0	11	0-50 kyr BP
M17049	Rosell-Melé et al.[26]	North Atlantic	freshening of surface waters in response to incoming freshwater from sea-ice and/or icebergs	55.3	-26.7	12	0-50 kyr BP
U1313	Naafs et al.[27]	North Atlantic	influence of polar water	41.0	-33.0	8	5.0-70 kyr BP
BOFS 16K	Weaver et al.[28]	North Atlantic	There is no advantage of including $C_{37:4}$ in SST reconstruction	59.0	-23.0	17	0-120 kyr BP
MD01-2412	Harada et al.[29]	Okhotsk Sea	low SSS due to declining supply of saline water /strengthening freshwater supply from the river/ increasing precipitation /effect of melting sea ice	44.5	145.0	69	0-115 kyr BP
PC-2	Seki et al.[30]	Okhotsk Sea	low salinity	50.4	148.3	35	0-85 kyr BP
ODP 977A	Martrat et al.[31]	west Mediterrania n	low salinity during Heinrich events	36.0	-2.0	10	0-250 kyr BP
MD01-2408	Fujine et al.[32]	Japan Sea	U^K_{37} is more suitable than $U^{K\prime}_{37}$	39.6	139.4	20	0-170 kyr BP
MD01-2444	Martrat et al.[33]	Iberian margin	advection of cold surface water	37.6	-10.1	13	135.1-185.7 kyr BP
PC05-21	Lee et al.[34]	Japan Sea	unclear	38.4	131.5	25	0-200 kyr BP
PS75/034 -2	Ho et al.[35]	Southern Ocean	U^K_{37} is more suitable than $U^{K\prime}_{37}$	-54.4	-80.1	23	0-700 kyr BP
U1385	Rodrigues et al.[36] Bajo et al.[37]	Iberian margin	freshwater input from melting iceberg during glacial extreme cold events	37.6	-10.1	15	0-1 Ma
U1341	Horikawa et al.[38]	Bering Sea	colder and fresher water	54.0	179.0	20	0-4.3 Ma
ODP 983C	McClymont et al.[39]	North Atlantic	polar water mass	60.4	-23.6	24	0.5-1.5 Ma
ODP 882	Martínez-Garcia et al.[40]	Sub-Arctic Pacific	polar/subpolar extent	50.4	167.6	28	0-3.5 Ma
ODP 1090	Martínez-Garcia et al.[40]	sub-Antarctic Atlantic	polar/subpolar extent	-42.9	8.9	21	0-3.5 Ma
U1417	Sánchez-Montes et al.[41]	Gulf of Alaska	cold and/or freshwater	57.0	-147.1	24	1.7-4 Ma
104-642B	Bachem et al.[42]	Norwegian Sea	cooler (potentially less saline) surface water	67.2	2.9	8	3.14-5.32 Ma
ACEX	Stein et al.[43]	Arctic	decrease in salinity (melting of sea ice and/or icebergs during summer?)	87.9	137.5	10	37-48 Ma
HM83-06	Rosell-Melé et al.[44]	North Sea Fan	-	64.3	-2.7	8	-
HM79-08	Rosell-Melé et al.[44]	North Sea Fan	-	62.9	0.0	8	-
162-986B	Rosell-Melé et al.[44]	Svalbard margin	-	77.3	-9.1	24	-

Supplementary Table 2. List of Canadian Arctic Archipelago surface sediment samples used in DNA sequencing and the amplicon sequence variants (ASVs) resolved.

Sample	Lat.	Lon.	ASV1	ASV2	ASV3	ASV4
QMG1	68.49	-99.89	0	34	0	0
QMG2	68.31	-100.80	0	0	20	0
312	69.17	-100.70	121	45	0	17

Supplementary References

1. Zheng, Y., Heng, P., Conte, M. H., Vachula, R. S. & Huang, Y. Systematic chemotaxonomic profiling and novel paleotemperature indices based on alkenones and alkenoates: Potential for disentangling mixed species input. *Org. Geochem.* **128**, 26–41 (2019).
2. Harada, N., Shin, K. H., Murata, A., Uchida, M. & Nakatani, T. Characteristics of alkenones synthesized by a bloom of *Emiliania huxleyi* in the Bering Sea. *Geochim. Cosmochim. Acta* **67**, 1507–1519 (2003).
3. Bendle, J., Rosell-Melé, A. & Ziveri, P. Variability of unusual distributions of alkenones in the surface waters of the Nordic seas. *Paleoceanography* **20**, PA2001 (2005).
4. Zweng, M. M. *et al*. World Ocean Atlas 2018, Vol. 2: Salinity. *NOAA Atlas NESDIS* **82**, 50 (2018)
5. Enberg, S., Majaneva, M., Autio, R., Blomster, J. & Rintala, J. Phases of microalgal succession in sea ice and the water column in the Baltic Sea from autumn to spring. *Mar. Ecol. Prog. Ser.* **599**, 19–34 (2018).
6. Martrat, B., Grimalt, J. O., Villanueva, J., van Kreveld, S. & Sarnthein, M. Climatic dependence of the organic matter contributions in the north eastern Norwegian Sea over the last 15,000 years. *Org. Geochem.* **34**, 1057–1070 (2003).
7. Johnsen, S. J. *et al.* Oxygen isotope and palaeotemperature records from six Greenland ice-core stations: Camp Century, Dye-3, GRIP, GISP2, Renland and NorthGRIP. *J. Quat. Sci.* **16**, 299–307 (2001).
8. Sarnthein, M. *et al.* Centennial-to-millennial-scale periodicities of Holocene climate and sediment injections off the western Barents shelf, 75°N. *Boreas* **32**, 447–461 (2003).
9. Perner, K. *et al.* An oceanic perspective on Greenland's recent freshwater discharge since 1850. *Sci. Rep.* **9**, 1-10 (2019).
10. Rueda, G., Fietz, S. & Rosell-Melé, A. Coupling of air and sea surface temperatures in the eastern Fram Strait during the last 2000 years. *The Holocene* **23**, 692–698 (2013).
11. Lochte, A. A. *et al.* Surface and subsurface Labrador Shelf water mass conditions during the last 6000 years. *Clim. Past* **16**, 1127–1143 (2020).
12. Krossa, V. R., Moros, M., Blanz, T., Jansen, E. & Schneider, R. Late Holocene Baltic Sea outflow changes reconstructed using $C_{37:4}$ content from marine cores: Late Holocene Baltic Sea outflow changes. *Boreas* **44**, 81–93 (2015).

13. Moros, M. *et al.* Surface and sub-surface multi-proxy reconstruction of middle to late Holocene palaeoceanographic changes in Disko Bugt, West Greenland. *Quat. Sci. Rev.* **132**, 146–160 (2016).
14. Rigual-Hernández, A. S. *et al.* Svalbard ice-sheet decay after the Last Glacial Maximum: New insights from micropalaeontological and organic biomarker paleoceanographical reconstructions. *Palaeogeogr. Palaeoclimatol. Palaeoecol.* **465**, 225–236 (2017).
15. Lochte, A. A. *et al.* Holocene water mass changes in the Labrador Current. *The Holocene* **29**, 676–690 (2019).
16. Bendle, J. A. P. & Rosell-Melé, A. High-resolution alkenone sea surface temperature variability on the North Icelandic Shelf: implications for Nordic Seas palaeoclimatic development during the Holocene. *The Holocene* **17**, 9–24 (2007).
17. Harada, N. *et al.* Deglacial–Holocene environmental changes at the Pacific entrance of the Strait of Magellan. *Palaeogeogr. Palaeoclimatol. Palaeoecol.* **375**, 125–135 (2013).
18. Ishiwatari, R., Houtatsu, M. & Okada, H. Alkenone-sea surface temperatures in the Japan Sea over the past 36 kyr: warm temperatures at the last glacial maximum. *Org. Geochem.* **32**, 57–67 (2001).
19. Kristjánsdóttir, G. B., Moros, M., Andrews, J. T. & Jennings, A. E. Holocene Mg/Ca, alkenones, and light stable isotope measurements on the outer North Iceland shelf (MD99-2269): A comparison with other multi-proxy data and sub-division of the Holocene. *The Holocene* **27**, 52–62 (2017).
20. Risebrobakken, B., Moros, M., Ivanova, E. V., Chistyakova, N. & Rosenberg, R. Climate and oceanographic variability in the SW Barents Sea during the Holocene. *The Holocene* **20**, 609–621 (2010).
21. Łącka, M. *et al.* Postglacial paleoceanography of the western Barents Sea: Implications for alkenone-based sea surface temperatures and primary productivity. *Quat. Sci. Rev.* **224**, 105973 (2019).
22. Calvo, E., Grimalt, J. & Jansen, E. High resolution U^{K}_{37} sea surface temperature reconstruction in the Norwegian Sea during the Holocene. *Quat. Sci. Rev.* **21**, 1385–1394 (2002).
23. Ménot, G. *et al.* Early Reactivation of European Rivers During the Last Deglaciation. *Science* **313**, 1623–1625 (2006).

24. Caniupán, M. *et al.* Millennial-scale sea surface temperature and Patagonian Ice Sheet changes off southernmost Chile (53°S) over the past ~60 kyr. *Paleoceanography* **26**, PA3221 (2011).
25. Roberts, J. *et al.* Deglacial changes in flow and frontal structure through the Drake Passage. *Earth Planet. Sci. Lett.* **474**, 397–408 (2017).
26. Rosell-melé, A. Appraisal of a molecular approach to infer variations in surface ocean freshwater inputs into the North Atlantic during the last glacial. *Glob. Planet. Change* **34**, 143–152 (2002).
27. Naafs, B. D. A. *et al.* Strengthening of North American dust sources during the late Pliocene (2.7Ma). *Earth Planet. Sci. Lett.* **317**, 8–19 (2012).
28. Weaver, P. P. E. *et al.* Combined coccolith, foraminiferal, and biomarker reconstruction of paleoceanographic conditions over the past 120 kyr in the northern North Atlantic (59°N, 23°W). *Paleoceanography* **14**, 336–349 (1999).
29. Harada, N., Sato, M. & Sakamoto, T. Freshwater impacts recorded in tetraunsaturated alkenones and alkenone sea surface temperatures from the Okhotsk Sea across millennial-scale cycles. *Paleoceanography* **23**, PA3201 (2008).
30. Seki, O. Decreased surface salinity in the Sea of Okhotsk during the last glacial period estimated from alkenones. *Geophys. Res. Lett.* **32**, L08710 (2005).
31. Martrat, B. *et al.* Abrupt Temperature Changes in the Western Mediterranean over the Past 250,000 Years. *Science* **306**, 1762–1765 (2004).
32. Fujine, K., Yamamoto, M., Tada, R. & Kido, Y. A salinity-related occurrence of a novel alkenone and alkenoate in Late Pleistocene sediments from the Japan Sea. *Org. Geochem.* **37**, 1074–1084 (2006).
33. Martrat, B. *et al.* Four Climate Cycles of Recurring Deep and Surface Water Destabilizations on the Iberian Margin. *Science* **317**, 502–507 (2007).
34. Lee, K. E., Bahk, J. J. & Choi, J. Alkenone temperature estimates for the East Sea during the last 190,000 years. *Org. Geochem.* **39**, 741–753 (2008).
35. Ho, S. L. *et al.* Sea surface temperature variability in the Pacific sector of the Southern Ocean over the past 700 kyr. *Paleoceanography* **27**, PA4202 (2012).

36. Rodrigues, T. *et al.* A 1-Ma record of sea surface temperature and extreme cooling events in the North Atlantic: A perspective from the Iberian Margin. *Quat. Sci. Rev.* **172**, 118–130 (2017).
37. Bajo, P. *et al.* Persistent influence of obliquity on ice age terminations since the Middle Pleistocene transition. *Science* **367**, 1235–1239 (2020).
38. Horikawa, K. *et al.* Pliocene cooling enhanced by flow of low-salinity Bering Sea water to the Arctic Ocean. *Nat. Commun.* **6**, 1-9 (2015).
39. McClymont, E. L., Rosell-Melé, A., Haug, G. H. & Lloyd, J. M. Expansion of subarctic water masses in the North Atlantic and Pacific oceans and implications for mid-Pleistocene ice sheet growth. *Paleoceanography* **23**, PA4214 (2008).
40. Martinez-Garcia, A., Rosell-Melé, A., McClymont, E. L., Gersonde, R. & Haug, G. H. Subpolar Link to the Emergence of the Modern Equatorial Pacific Cold Tongue. *Science* **328**, 1550–1553 (2010).
41. Sánchez-Montes, M. L. *et al.* Late Pliocene Cordilleran Ice Sheet development with warm northeast Pacific sea surface temperatures. *Clim. Past* **16**, 299–313 (2020).
42. Bachem, P. E., Risebrobakken, B., De Schepper, S. & McClymont, E. L. Highly variable Pliocene sea surface conditions in the Norwegian Sea. *Clim. Past* **13**, 1153–1168 (2017).
43. Stein, R. Blackman, D., Inagaki, F., & Larsen, H.C. *Earth and Life Processes Discovered from Subseafloor Environments - A Decade of Science Achieved by the Integrated Ocean Drilling Program (IODP)*. vol. 7 (Elsevier, 2014).
44. Rosell-Melé, A. *et al.* Alkenones and coccoliths in ice-rafted debris during the Last Glacial Maximum in the North Atlantic: implications for the use of U^{K}_{37}' as a sea surface temperature proxy. *J. Quat. Sci.* **26**, 657–664 (2011).
