# Supplementary material for: Group 2i Isochrysidales produce characteristic alkenones reflecting sea ice distribution
Source: Nat Commun. 2021 Jan 4;12:15. doi: 10.1038/s41467-020-20187-z (PMC7782803; doi:10.1038/s41467-020-20187-z)
Supplement: Supplementary file 5 — Description of Additional Supplementary Files [file 41467_2020_20187_MOESM5_ESM.pdf]

Description of Additional Supplementary Information

File Title: Supplementary Dataset 1

Description: List of surface sediment samples analyzed for alkenones

File Title: Supplementary Dataset 2

Description: List of re-analyzed NGS datasets and the Isochrysidales sequences detected
