# Supplementary material for: Group 2i Isochrysidales produce characteristic alkenones reflecting sea ice distribution
Source: Nat Commun. 2021 Jan 4;12:15. doi: 10.1038/s41467-020-20187-z (PMC7782803; doi:10.1038/s41467-020-20187-z)
Supplement: Supplementary file 6 — Reporting Summary [file 41467_2020_20187_MOESM6_ESM.pdf]

nature research

Corresponding author(s): Karen Wang
Yongsong Huang

Last updated by author(s): Nov-11-2020

Reporting Summary

Nature Research wishes to improve the reproducibility of the work that we publish. This form provides structure for consistency and transparency in reporting. For further information on Nature Research policies, see our Editorial Policies and the Editorial Policy Checklist.

Statistics

For all statistical analyses, confirm that the following items are present in the figure legend, table legend, main text, or Methods section.

n/a	Confirmed	
☐	☒	The exact sample size (*n*) for each experimental group/condition, given as a discrete number and unit of measurement
☐	☒	A statement on whether measurements were taken from distinct samples or whether the same sample was measured repeatedly
☒	☐	The statistical test(s) used AND whether they are one- or two-sided<br>*Only common tests should be described solely by name; describe more complex techniques in the Methods section.*
☐	☒	A description of all covariates tested
☒	☐	A description of any assumptions or corrections, such as tests of normality and adjustment for multiple comparisons
☐	☒	A full description of the statistical parameters including central tendency (e.g. means) or other basic estimates (e.g. regression coefficient) AND variation (e.g. standard deviation) or associated estimates of uncertainty (e.g. confidence intervals)
☐	☒	For null hypothesis testing, the test statistic (e.g. F, t, r) with confidence intervals, effect sizes, degrees of freedom and P value noted<br>*Give P values as exact values whenever suitable.*
☒	☐	For Bayesian analysis, information on the choice of priors and Markov chain Monte Carlo settings
☒	☐	For hierarchical and complex designs, identification of the appropriate level for tests and full reporting of outcomes
☒	☐	Estimates of effect sizes (e.g. Cohen's *d*, Pearson's *r*), indicating how they were calculated

Our web collection on statistics for biologists contains articles on many of the points above.

Software and code

Policy information about availability of computer code

Data collection	Alkenone data were collected through Agilent ChemStation B.03.02
Data analysis	Statistical analyses were performed via MATLAB (R2018b). Softwares used in DNA analysis includes: FastQC v0.11.8, UNOISE3, QIIME/1.9.1, DADA2, PyNast 1.0, Cutadapt 1.9. Phylogenetic analysis were performed via RAxML v8.2.12 on CIPRES Science Gateway V 3.3.

For manuscripts utilizing custom algorithms or software that are central to the research but not yet described in published literature, software must be made available to editors and reviewers. We strongly encourage code deposition in a community repository (e.g. GitHub). See the Nature Research guidelines for submitting code & software for further information.

Data

Policy information about availability of data

All manuscripts must include a data availability statement. This statement should provide the following information, where applicable:

- Accession codes, unique identifiers, or web links for publicly available datasets
- A list of figures that have associated raw data
- A description of any restrictions on data availability

Raw sequence data generated in this study were archived in NCBI GenBank with accession number PRJNA650544, assembled Isochrysidales sequences were archived in NCBI GenBank with accession number MT647637-MT647640. Re-analyzed NGS data can be found through European Nucleotide Archive (ENA) with accession codes provided in Supplementary Data 2. Reference database SILVA is available at https://www.arb-silva.de/, and PR2 is available at https://github.com/pr2database/pr2database.

Field-specific reporting

Please select the one below that is the best fit for your research. If you are not sure, read the appropriate sections before making your selection.

☒ Life sciences ☐ Behavioural & social sciences ☐ Ecological, evolutionary & environmental sciences

For a reference copy of the document with all sections, see nature.com/documents/nr-reporting-summary-flat.pdf

Life sciences study design

All studies must disclose on these points even when the disclosure is negative.

Sample size	No sample-size calculation were performed. For the algal culture experiments, we are confident that the alkenones produced by each culture sample were representative as the alkenone concentrations were sufficiently high. For DNA sequencing of the surface sediment samples, the sample size was determined by sample availability.
Data exclusions	No data were excluded from the study.
Replication	Each culture experiment were replicated three times, all replicates are successful and included. DNA sequencing of surface sediment samples were not replicated due to sample availability, as we had to ensure enough sample weight for organic geochemical analysis.
Randomization	For all the culture experiments, samples were randomly assigned to different temperature and salinity conditions. Randomization was not relevant in DNA sequencing as all sediment samples had the same treatment.
Blinding	Blinding was not relevant in the study, as both the culture and DNA sequencing data are objectively derived from lab instruments.

Reporting for specific materials, systems and methods

We require information from authors about some types of materials, experimental systems and methods used in many studies. Here, indicate whether each material, system or method listed is relevant to your study. If you are not sure if a list item applies to your research, read the appropriate section before selecting a response.

Materials & experimental systems

n/a	Involved in the study	
☒	☐	Antibodies
☒	☐	Eukaryotic cell lines
☒	☐	Palaeontology and archaeology
☒	☐	Animals and other organisms
☒	☐	Human research participants
☒	☐	Clinical data
☒	☐	Dual use research of concern

Methods

n/a	Involved in the study	
☒	☐	ChIP-seq
☒	☐	Flow cytometry
☒	☐	MRI-based neuroimaging
